# Blood glucose lowering activity of aloe based composition, UP780, in alloxan induced insulin dependent mouse diabetes model

**DOI:** 10.1186/1758-5996-6-61

**Published:** 2014-05-24

**Authors:** Mesfin Yimam, Jifu Zhao, Brandon Corneliusen, Mandee Pantier, Lidia Brownell, Qi Jia

**Affiliations:** 1 Unigen, Inc, 3005 1st Ave, Seattle, WA, 98121, USA

**Keywords:** Insulin resistance, Aloe vera, Chromones, Alloxan

## Abstract

**Background:**

There are a few nutritional approaches to address the increased needs of managing diabetic conditions. Previously it has been reported that UP780, a standardized composition of aloe chromone formulated with an aloe polysaccharide, has a significant impact in reducing HbA1C, fasting blood glucose, fructosamine and plasma insulin level in humans and improved impaired glucose and insulin resistance in high-fat diet-induced and db/db non-insulin dependent diabetic mouse models. Here we describe activity of UP780 and its constituents to improve insulin sensitivity in alloxan induced insulin dependent diabetic mouse model.

**Materials and method:**

Insulin dependent diabetes was induced by administering a single intraperitoneal injection of alloxan monohydrate at a dose of 150 mg/kg to CD-1 mice. Aloesin (UP394) was formulated with an Aloe vera inner leaf gel powder polysaccharide (Qmatrix) to yield a composition designated UP780. Efficacy of oral administration of UP780 at 2000 mg/kg and its constituents (aloesin at 80 mg/kg and Qmatrix at 1920 mg/kg) were evaluated in this model. Glyburide, a sulfonylurea drug used in the treatment of type 2 diabetes, was used at 5 mg/kg as a positive control. Effect of UP780 on non-diabetic normal mice was also addressed.

**Results:**

Mice administered intraperitoneal alloxan monohydrate developed progressive type-1 diabetes like symptom. After 4 weeks of daily oral administration, reductions of 35.9%, 17.2% and 11.6% in fasting blood glucose levels were observed for UP780, the UP780 Aloe vera inner leaf gel polysaccharide preparation without chromone (Qmatrix), and Aloesin (UP394), treated animals respectively, compared to vehicle treated animals. UP780 has no impact on blood glucose level of non-diabetic healthy mice. UP780 showed statistically significant improvement for blood glucose clearance in oral glucose tolerance tests. Similarly, enhanced improvement in plasma insulin level and statistically significant reduction in triglyceride level was also observed for animals treated with the composition.

**Conclusion:**

These findings suggest that UP780, a chromone standardized Aloe based composition, could possibly be used as a natural supplement alternative to facilitate maintenance of healthy blood glucose levels.

## Introduction

Ethnobotanical archives reveal that more than 800 plants have been used as an alternative remedies for the treatment of high blood glucose associated human ailments with minor or no scientific evidence. It’s not uncommon to find Aloe as an alternative solution in this front line for many years. Aloe is a biochemically complex plant that contains many biologically active substances [1]. Historically, Aloe products have been used in dermatological applications for the treatment of burns, sores and wounds. These uses have stimulated a great deal of research in identifying polysaccharides, chromones, anthraquinones and other compounds from Aloe plants that have clinical implications as anti-inflammatory [2,3] anti-tumor, anti-gastric ulcer, anti-diabetic, anti-tyrosinase [4] and antioxidant activity [5].

*Aloe vera,* also with a Latin name *Aloe barbadensis,* leaves and their bitter principles exhibit effects on blood glucose level in normal and alloxan-induced diabetic mice [6], and dried sap of various Aloe species demonstrates anti-diabetic activity in clinical studies [7].

**Figure 1:**
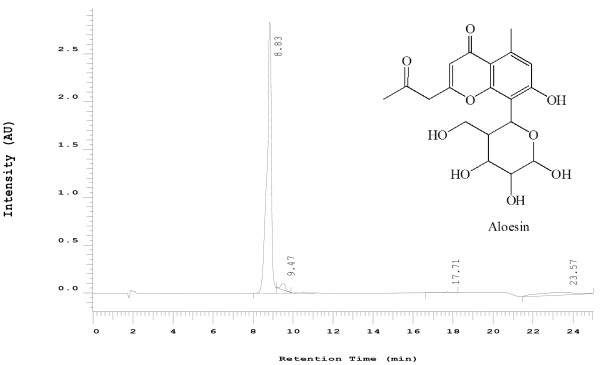
HPLC chromatograph of Aloesin (UP394).

Chromones are a specific type of aromatic compounds having a benzopyran-4-one as their major skeletal structure as illustrated by the Figure 1. Chomones isolated from various Aloe species have been reported to have diverse biological activity. Aloesin have reportedly inhibited tyrosinase activity [8] and up-regulate cyclin E-dependent kinase activity [9]. Those chromones isolated from *Aloe barbadensis* also demonstrate anti-inflammatory [10] and antioxidant activity [11].

A large diversity of animal models has been developed to better understand the pathogenesis of insulin dependent diabetes and new drugs have been introduced into the market to treat the primary cause or to prevent the catastrophe of its complications. Among these models, alloxan induced diabetes model is by far the most frequently used model which has been useful for the study of multiple aspect of the disease [12,13].

Alloxan, as a cytotoxic agent to the insulin-secreting β cells of the pancreas, effectively induces insulin dependent phenotypes that resemble type 1 diabetes or post-beta cell “burnout” type 2 diabetes in a wide variety of animal models [12,13]. Thus, it allows elucidation of antihyperglycemic agents in the treatment of diabetes. Alloxan-induced diabetes consistently produced the main characteristics of diabetes mellitus including polydipsia, polyphagia, polyuria, decreased insulin levels, weight loss and hyperglycemia.

Though lifestyle intervention, mainly diet and exercise, remain as the ultimate behavioral change in managing metabolic syndrome and diabetic conditions; it is desirable to have safe nutritional approaches for preventing and/or ameliorating insulin resistance. In this frontier use of dietary supplements could be a potential option to control diabetic conditions. To date, many plant derived extracts have been studied, reported and commercialized for glucose lowering effects. However, very few of them are supported by credible chemical profiles, pre-clinical and clinical trial data to support associated claims.

The present study was therefore designed to evaluate a standardized composition, UP780, an aloe chromone formulated with an aloe polysaccharide, improves insulin sensitivity in alloxan induced insulin dependent diabetic mice.

## Materials and methods

### Animals and housing

Purpose bred female CD-1 mice, weighing 18-24 g, were purchased from USDA approved laboratory animal vendor (Charles River Laboratories, Inc., Wilmington, MA) and acclimated upon arrival for a week. Individual cages were identified with a cage card indicating project number, test article, dose level, group, and animal number. The Harlan T7087 soft cob beddings was used and changed at least twice/week. Animals were provided with fresh water and rodent chow diet # T2018 (Harlan Teklad, 370 W, Kent, WA) ad libitum and were housed in a temperature controlled room (22.2°C) on a 12 hour light-dark cycle. All animal experiments were conducted according to institutional guidelines congruent with the guide for the care and use of laboratory animals. All animal experiments were conducted according to institutional guidelines congruent with the guide for the care and use of laboratory animals. IACUC approval reference #: UAS-101205.

### Model induction and dosing

To induce disease model, overnight fasted mice received a single injection of alloxan monohydrate (Sigma Chemical, St. Louis, OH) at a dose of 150 mg/kg body weight intraperitoneally [12,13]. The induction of alloxan-induced diabetes was confirmed by measuring blood glucose level on the 5^th^ day after alloxan administration. Mice with glucose levels above 200 mg/dl were selected for randomized grouping. Average blood glucose levels for the groups ranged between 368 – 389 mg/dl. Two group of mice (n = 15/group) which did not receive alloxan served as the non-diabetic control (one group treated with vehicle and the other group treated with UP780 at 2000 mg/kg).

Once an induction of the disease was confirmed and randomization was completed, daily oral treatment was initiated. Fresh test compounds and positive control were prepared each day and delivered orally to mice (N = 15) at a dose of 2000 mg/kg standardized composition (UP780), 1920 mg/kg Aloe vera inner leaf gel powder polysaccharide (Qmatrix) and 80 mg/kg chromone aloesin (UP394) (Figure 1). Previously we have shown 200 mg/kg as the effective dose of UP780 in high fat diet induced diabetic mice. 10 times the effective dose (2000 mg/kg) were selected in the current study to evaluate the impact of UP780 in the blood glucose level of diseased and normal mice. The composition UP780, comprised of 4% aloesin and 96% aloe vera and hence a dose of 80 mg/kg and 1920 mg/kg, respectively, were used for this study. The positive control group received 5 mg/kg Glyburide. Treatment lasted for 4 weeks. Research grade de-ionized water was used for suspending test compounds. The positive control, Glyburide, was first dissolved in DMSO and mixed with 0.25% of methylcellulose (Spectra, Lot# 116H0857) and 0.5% of Tween 20 (Sigma, Lot # 036 K00961) at 10%. Test compounds were repeatedly vortexed between gavages to maintain consistent dosing. The carrier vehicle treated animals received de-ionized water only. No detectable sign of irritation was observed from drug or vehicle administration.

### Fasting glucose, triglyceride and plasma insulin

Fasting blood glucose and triglyceride were taken at pre-induction, week-0 (baseline), week-2 and week-4 after treatment of overnight fasted mice. Plasma insulin levels were determined at pre-induction, baseline (week-0) and week-4.

### Oral glucose tolerance test (OGTT)

Oral glucose tolerance test (OGTT) was conducted 4-week after onset of treatment [14,15]. On the test day, animals were fasted for 5 hours and received oral administration of glucose at a dose of 2 g/kg. Blood glucose levels were determined at time 0 (before glucose injection), and at 30, 60, 90, and 120 minutes post glucose delivery. Blood samples were obtained from the tail

### Assays

Blood glucose levels were measured using the IQ blood glucose monitor with prestige test strips (Walgreen, Home Diagnostics, Inc., Ft. Lauderdale, FL). Total triglyceride levels were measured using the CardioChek Analyzer with PTS panels test strips (Polymer Technology System, Inc, Indianapolis, IN). Plasma insulin levels were measured with an ELISA kit and accompanied protocol for insulin (Crystal Chem - Chicago, IL). In brief, a mouse insulin ELISA kit was purchased from Crystal Chemical (Cat# 90080) to test the insulin content of mouse plasma. The provided sample diluent (95 μL) was dispensed into each well of a 96-well antibody-coated microplate. Samples and insulin standards (5 μL) were added to the wells in duplicate and plates were incubated for 2 hours at 4°C. Each well was then washed five times with the provided wash buffer. The anti-insulin enzyme conjugate (100 μL) was added to each well and the microplate was incubated at room temperature for 30 minutes. Each well was washed seven times with the provided wash buffer and the enzyme substrate (100 μL) was then added to each well. The microplate was incubated at room temperature for 40 minutes before adding enzyme reaction stop solution (100 μL) to each well. Each plate was read on a Wallac Victor^2^ microplate reader at A_450_ with A_570_ values subtraction. Insulin concentration of the samples was calculated using the standard curve created by the insulin standards. Blood was collected by tail vein and spun down for plasma.

### Statistical analysis

Data were analyzed using Sigmaplot (Version 11.0). The results are represented as mean ± one S.D. Statistical significance between groups was calculated by means of single factor analysis of variance followed by a paired *t*-test. *P*-values less or equal to 0.05 (P ≤ 0.05) were considered as significant. When normality test failed, for non-parametric analysis, data were subjected to Mann-Whitney sum ranks for *t*-test and Kruskal-Wallis one way analysis of variance on ranks for ANOVA. A conventional trapezoid rule was used to determine area under the curve (AUCt_0-120_) for glucose tolerance test.

## Results

**Figure 2:**
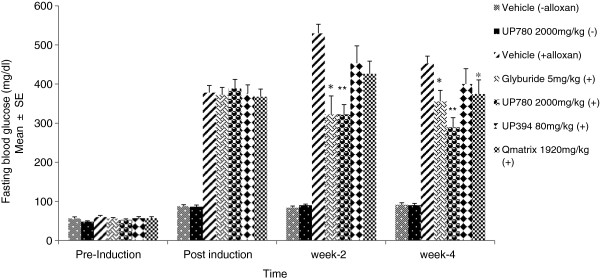
**Fasting blood glucose level.** Insulin dependent diabetes was induced by administering a single intraperitoneal injection of alloxan monohydrate at a dose of 150 mg/kg to CD-1 mice. Mice (n = 15/group) were fasted overnight before measurements were taken for fasting blood glucose levels at pre-induction, week-0 (baseline), week-2 and week-4 after treatment. Data are expressed as mean ± SD. * P ≤ 0.05.

Mice administered a single intraperitoneal injection of alloxan monohydrate at a dose of 150 mg/kg showed progression of type-I diabetes symptoms (Figure 2). As seen from the data in Figure 2, fasting blood glucose levels were reduced versus vehicle treatment following two weeks of oral treatment with Glyburide (39.1% reduction), UP780 (39.1% reduction), aloesin (14.5% reduction), and Qmatrix (19.6% reduction). Similarly, after 4-weeks of oral treatment with Glyburide, UP780, UP394 and Qmatrix, a 21.4%, 35.9%, 11.6% and 17.2% reduction in fasting blood glucose was measured for each respective treatment group compared to the vehicle control group. Only UP780 and glyburide showed statistically significant reduction in blood glucose level at both weeks 2 (P = 0.00001, P = 0.003, respectively) and week-4 (P = 0.0001, P = 0.01, respectively). Qmatrix treated animals showed statistically significant decrease in blood glucose level at week 2 (P = 0.01). Fasting blood glucose levels for UP780 or vehicle treated non-diabetic normal mice, i.e., without alloxan administered, were unaffected at each time points monitored.

**Figure 3:**
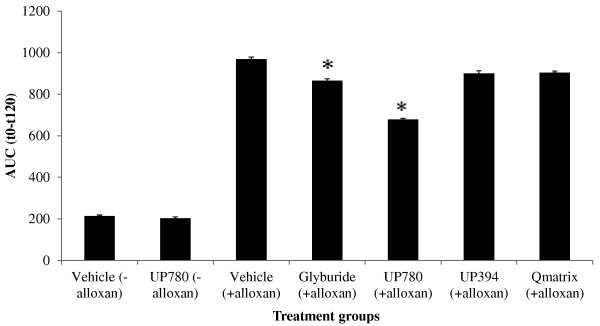
**Area-under-concentration curve value of glucose in oral glucose tolerance test (AUC **_**(t0-t120)**_**).** Oral glucose tolerance test (OGTT) was conducted 4-week after onset of treatment. On the test day, animals (N = 15/group) were fasted for 5 hours and received oral administration of glucose at a dose of 2 g/kg. Blood glucose levels were determined at time 0 (before glucose injection), and at 30, 60, 90, and 120 minutes post glucose delivery. Blood samples were obtained from the tail. Data are expressed as mean ± SD. A conventional trapezoid rule was used to determine area under the curve (AUCt_0-120_) for glucose tolerance test. * P ≤ 0.05.

From oral glucose tolerance test (OGTT) data, it was observed that mice treated with 2000 mg/kg of UP780 or 5 mg/kg glyburide showed significantly improved clearance of blood glucose at each time point monitored versus vehicle treated diabetic mice (Table 1). Other treatment groups showed similar blood glucose clearance to that of vehicle after oral glucose load. Normal mice treated with vehicle or UP780 showed equivalent glucose utilization that was superior to all other treatment groups. As illustrated in area-under-the curve (AUC_t0-t120_) of blood glucose levels from the OGTT data, UP780 has statistically significant low levels of blood glucose than either aloesin or Qmatrix (Figure 3). Similarly statistical significant reductions in total triglyceride level were observed both at week-2 and week-4 for mice treated with UP780. However, these triglyceride reductions were statistically significant only at week-2 for aloesin and week-4 for Qmatrix. In contrast, the values were unaffected for non-diabetic mice treated either vehicle or UP780 (Table 2).

**Table 1:** Oral glucose tolerance test blood glucose values

**Blood glucose (mg/dl)**						
**Group**	**Dose (mg/kg)**	**T0**	**T30**	**T60**	**T90**	**T120**
Vehicle (-alloxan)	0	91.9 ± 4.9	125.2 ± 5.3	114.3 ± 6.4	93.4 ± 7.9	98.3 ± 5.4
UP780 (-)	2000	90.5 ± 4.2	113.7 ± 5.9	108.5 ± 4.0	95.1 ± 4.1	85.3 ± 3.3
Vehicle (+alloxan)	0	452.6 ± 18.9	518.0 ± 18.1	492.0 ± 22.6	486.1 ± 23.6	431.9 ± 21.2
Glyburide (+)	5	355.8 ± 21.6*	472.7 ± 28.2	438.5 ± 28.4	441.7 ± 35.1	398.7 ± 19.1
UP780 (+)	2000	289.9 ± 24.2*	382.9 ± 21.0*	331.2 ± 33.0*	339.7 ± 34.5*	315.1 ± 34.9*
UP394 (+)	80	400.2 ± 39.0	432.9 ± 33.0	483.4 ± 44.3	462.9 ± 47.5	442.1 ± 48.0
Qmatrix (+)	1920	374.7 ± 36.0	494.4 ± 32.0	447.5 ± 36.6	458.2 ± 43.0	443.2 ± 42.0

Insulin dependent diabetes was induced by administering a single intraperitoneal injection of alloxan monohydrate at a dose of 150 mg/kg to CD-1 mice. Oral glucose tolerance test (OGTT) was conducted 4-week after onset of treatment. On the test day, animals (N = 15/group) were fasted for 5 hours and received oral administration of glucose at a dose of 2 g/kg. Blood glucose levels were determined at time 0 (before glucose injection), and at 30, 60, 90, and 120 minutes post glucose delivery. Blood samples were obtained from the tail. Data are expressed as mean ± SD. * P ≤ 0.05.

**Table 2:** Fasting blood Triglyceride level

**Group**	**Dose (mg/kg)**	**Triglyceride (mg/dl)**			
		**Pre-induction**	**Post-induction**	**Week-2**	**Week-4**
		**(Mean + SE)**	**(Mean + SE)**	**(Mean + SE)**	**(Mean + SE)**
Vehicle (-alloxan)	0	125.1 ± 10.7	90.1 ± 3.7	119.7 ± 4.6	126.9 ± 10.6
UP780 (-)	2000	118.1 ± 8.5	92.3 ± 3.6	128.7 ± 6.4	110.5 ± 5.5
Vehicle (+alloxan)	0	141.1 ± 7.3	111.5 ± 37.6	167.8 ± 20.0	144.0 ± 14.2
Glyburide (+)	5	135.3 ± 32.0	134.6 ± 37.5	135.6 ± 48.4	163.1 ± 38.8
UP780 (+)	2000	133.5 ± 7.8	117.5 ± 10.0	124.0 ± 8.8*	124.3 ± 8.2*
UP394 (+)	80	135.2 ± 8.2	121.3 ± 16.1	116.6 ± 8.41*	128.2 ± ±11.7
Qmatrix (+)	1920	130.5 ± 10.1	101.4 ± 7.3	119.7 ± 17.1	113.8 ± 30.3*

Insulin dependent diabetes was induced by administering a single intraperitoneal injection of alloxan monohydrate at a dose of 150 mg/kg to CD-1 mice. Measurements for fasting blood triglyceride were taken at pre-induction, week-0 (baseline), week-2 and week-4 after treatment. Data are expressed as mean ± SD. *P ≤ 0.05.

Plasma insulin levels were also measured to assess disease progression and severity. As presented in Table 3, post alloxan-induction plasma insulin levels were found to be the same or lower than pre-induction plasma insulin levels across all treatment groups. After four weeks of treatment, a similar trend of increased plasma insulin level was observed for all treatment groups including vehicle except for aloesin treated animals. The aloesin treatment group at week-4 showed a 4.8% reduction in insulin levels from pre-treatment baseline values. However, this increase of plasma insulin level for alloxan-induced diabetic animals treated with glyburide and UP780 at week-4 was doubled to that observed in the vehicle plus alloxan control group.

**Table 3:** Plasma insulin level

**Plasma insulin (ng/ml)**					
**Group**	**Dose (mg/kg)**	**Pre-induction**	**Post-induction**	**Week-4**	**% change after treatment**
		**(Mean + SE)**	**(Mean + SE)**	**(Mean + SE)**	
Vehicle (-alloxan)	0	1.25 ± 0.27	1.23 ± 0.29	4.26 ± 0.36	246.3 ↑
UP780 (-)	2000	0.99 ± 0.14	0.99 ± 0.27	4.12 ± 0.39	316.2 ↑
Vehicle (+alloxan)	0	1.78 ± 0.27	1.50 ± 0.34	1.99 ± 0.24	32.7 ↑
Glyburide (+)	5	1.87 ± 0.33	1.10 ± 0.15	1.76 ± 0.50	60.0 ↑
UP780 (+)	2000	1.96 ± 0.31	1.48 ± 0.33	2.37 ± 0.41	60.1 ↑
UP394 (+)	80	2.48 ± 0.5	1.68 ± 0.48	1.60 ± 0.37	4.8 ↓
Qmatrix (+)	1920	1.89 ± 0.28	1.50 ± 0.21	1.69 ± 0.32	12.7 ↑

Insulin dependent diabetes was induced by administering a single intraperitoneal injection of alloxan monohydrate at a dose of 150 mg/kg to CD-1 mice. Measurements for plasma insulin were taken at pre-induction, week-0 (baseline), and week-4 after treatment. Data are expressed as mean ± SD as well as percent change of treatment. ↑ increase from post induction, ↓ decrease from post induction.

## Discussion

Significant reports have demonstrated the glucose lowering effect of Aloe plant in animal models. For instance, reductions in fasting blood glucose and triglyceride levels [16], improved liver gluconeogenesis and improved lipid profiles [17,18], decreased oxidative brain damage [19,20] decreased lipid peroxidation in kidney [21] and liver [22] in streptozotocin induced diabetic rats; improved insulin resistance and impaired glucose tolerance in high-fat diet-induced [23,24] and db/db non-insulin dependent diabetic mice [24,25] were reported.

In humans, hypoglycemic and hypolipidemic in Aloe vera gel preparation combined with psyllium seed husks [26], reduced blood glucose and triglyceride level in women treated with aloe vera gel alone [27] or men in combination with glibenclamide [28], lower fasting blood glucose levels when Aloe vera latex is given to non-insulin dependent diabetic patients [7], decreased levels of total serum cholesterol, triglycerides, and low-density lipoproteins when whole-leaf Aloe vera extract administered patients with hyperlipidemia [29], significant reductions in HbA1c, fructosamine, fasting glucose, and insulin when Aloe vera inner leaf gel powder standardized with 2% aloesin given to patients with prediabetes or metabolic syndrome [30] and anti-hyperglycemic and anti-hypercholesterolemic for hyperlipidemic type 2 diabetic patients in Aloe vera leaf gel [31] were reported.

It is very unusual though for either animal or human studies of natural ingredients in diabetes to adequately describe the composition used, including botanical species, plant part, active components and preparation, and to include an assessment of the dose. Similarly, activity of aloe as a glucose lowering agent could be affected by 1) processing 2) parts of the Aloe used 3) animal models selected and 4) structure and content of active components. Likewise some bioactive components such as chromones of Aloe are limited to the rinds of the plant and the manufacture processes usually removes them with other anthraquinone compounds [4]. Hence the current study was conducted to evaluate glucose lowering activity of a defined composition (UP780) of *Aloe vera* inner leaf gel powder (Qmatrix) with aloe chromones (aloesin) in insulin dependent alloxan induced type-1 diabetes model and non-diabetic healthy CD-1 mice.

Mice received a single injection of intraperitoneal alloxan monohydrate at a dose of 150 mg/kg per day showed type 1 diabetes phenotype. Both UP780 and glyburide improved fasting blood glucose levels and glucose tolerance, and increased insulin levels to a greater extent than the UP780 constituents, aloesin and Qmatrix.

Plasma insulin level was also measured to estimate degree of disease progression and severity in alloxan induced diabetes model. Post induction plasma insulin level was found lower than pre-induction across treatment groups including vehicle treated mice except for non-diabetic mice treated with vehicle or UP780. After 28 days of daily oral treatment, a similar trend in increase plasma insulin level was observed for all treatment groups including vehicle except UP394 treated animals that showed 4.8% reduction from baseline. An increase in plasma insulin levels was also seen for vehicle treated, though less than 50% that seen with UP780 and glyburide. This result with vehicle treated mice suggests regeneration of beta-cells or compensatory conversion of alpha-cells to produce insulin accounts for some of the increase in insulin [32].

The increase in plasma insulin level observed for UP780 treated non-diabetic mice was consistent to that of vehicle treated non-diabetic mice. In week 4, the plasma insulin level of mice with no alloxan injection was 7.5 - 9.6 fold higher than that of the vehicle control mice with alloxan. This surge of plasma insulin level could be the result of metabolic compensation occurred after fasting in response to hypoglycemia. However, neither UP780 nor vehicle affects blood glucose level of healthy mice. Substantiating our finding Okyar et al. [33], have reported the neutral effect of Aloe vera leaf pulp and gel extracts on blood glucose level of non-diabetic rats.

Possibly UP780 could partially incurred its activity in this model through protection and/or stimulation of existing beta-cells to produce increased level of insulin. Clearly interestingly though, an unexpected synergy was observed from the combination of aloesin with aloe polysaccharidein that the beneficial effects seen with UP780 treatment exceed that predicted based on simply summing the effects observed for each of its constituents. The reduction in fasting blood glucose level of diabetic mice in the present investigation clearly reveals UP780 possess potential antidiabetic activity. UP780, comprised of 4% aloesin and 96% aloe vera, could have assisted surviving β-cells to resist the oxidative damage or prevent further damage caused by alloxan. Alloxan, as a cytotoxic agent to the insulin-secreting β cells of the pancreas, effectively induces insulin dependent phenotypes that resemble type 1 diabetes or post-beta cell “burnout” type 2 diabetes. In a way, the daily administration of UP780 would lead to the presence of known anti-oxidants such as aloesin and aloe polysaccharides as a shield providing an antioxidant activity to the β-cells of the islets of Langerhans from the destruction caused by superoxide radicals derived from alloxan [10,11,16]. In addition, the possibility of glyburide enhancing β-cells responsiveness to glucose has also been reported [34]. This mechanism of action could also be another possibility of UP780 to alleviate hyperglycemia; however, further detailed investigation mainly focused on the mode of action of UP780 could shed a better understanding how the composition be better utilized in targeting the pathogenesis of diabetes. In fact some have suggested that hypoglycemic activity of aloe observed in alloxan induced insulin dependent mouse model could be associated with its radical scavenging activity [23,35-37] or stimulate synthesis and/or release of insulin from beta-cell of pancreases [6].

Parallel to our result, Ghannnam et al. [7] showed that glibenclamide and aloe have statistically significant impact in reducing fasting blood glucose level in diabetic mice induced by alloxan. In this study, diabetic mice were given 10 mg/kg and 500 mg/kg of glibenclamide and aloe, respectively, twice per day for 14 days. Fasting blood glucose level was found significantly lower for both treatment groups after 3 days of treatment; however, after a week only the aloe treated mice showed the significance. In another study conducted by Ajabnoor (1990) also showed that when aloe was given for 4 days at 500 mg/kg twice per day, significant reduction in fasting blood glucose level were observed in alloxan induced diabetic mouse.

## Conclusion

Clinically it has been reported that UP780 can result in a statistically significant reduction in HbA1C, fasting blood glucose, fructosamine and plasma insulin level [34]. In addition to the data depicted here, previously we have shown that UP780 could improve impaired glucose and insulin resistance in high-fat diet-induced and db/db non-insulin dependent diabetic mouse models. Therefore, UP780, a standardized Aloe chromone based composition, could possibly be considered as a natural supplement alternative to maintain healthy blood glucose level.

## Competing interests

All authors, except Mandee Pantier and Jifu Zhao, are currently Unigen employees, therefore with financial interests.

## Authors’ contributions

MY conceived and designed, carried out study, data calculation, statistical analysis, data interpretation, and drafted/edited the manuscript. MP assisted in conducting the study. BC carried out the ELISA assay for plasma insulin. JZ conducted structure elucidations and identification. QJ and LB conceived the study, participated in its design, interpreted data, and edited the manuscript. All authors read and approved the final manuscript.
